# On the origin of open-circuit voltage losses in flexible *n-i-p* perovskite solar cells

**DOI:** 10.1080/14686996.2019.1633952

**Published:** 2019-06-21

**Authors:** Stefano Pisoni, Martin Stolterfoht, Johannes Löckinger, Thierry Moser, Yan Jiang, Pietro Caprioglio, Dieter Neher, Stephan Buecheler, Ayodhya N. Tiwari

**Affiliations:** a Laboratory for Thin Films and Photovoltaics, Empa-Swiss Federal Laboratories for Materials Science and Technology, Duebendorf, Switzerland; b Institute of Physics and Astronomy, University of Potsdam, Potsdam-Golm, Germany; c Young Investigator Group Perovskite Tandem Solar Cells, Helmholtz-Zentrum Berlin für Materialien und Energie GmbH, Berlin, Germany

**Keywords:** Perovskite solar cell, flexible, interface engineering, non-radiative recombination, quasi-Fermi level splitting, 50 Energy Materials, 100 Materials; 201 Electronics / Semiconductor / TCOs, 206 Energy conversion / transport / storage / recovery, 209 Solar cell / Photovoltaics, 212 Surface and interfaces, 306 Thin film / Coatings

## Abstract

The possibility to manufacture perovskite solar cells (PSCs) at low temperatures paves the way to flexible and lightweight photovoltaic (PV) devices manufactured via high-throughput roll-to-roll processes. In order to achieve higher power conversion efficiencies, it is necessary to approach the radiative limit via suppression of non-radiative recombination losses. Herein, we performed a systematic voltage loss analysis for a typical low-temperature processed, flexible PSC in *n-i-p* configuration using vacuum deposited C_60_ as electron transport layer (ETL) and two-step hybrid vacuum-solution deposition for CH_3_NH_3_PbI_3_ perovskite absorber. We identified the ETL/absorber interface as a bottleneck in relation to non-radiative recombination losses, the quasi-Fermi level splitting (QFLS) decreases from ~1.23 eV for the bare absorber, just ~90 meV below the radiative limit, to ~1.10 eV when C_60_ is used as ETL. To effectively mitigate these voltage losses, we investigated different interfacial modifications via vacuum deposited interlayers (BCP, B4PyMPM, 3TPYMB, and LiF). An improvement in QFLS of ~30–40 meV is observed after interlayer deposition and confirmed by comparable improvements in the open-circuit voltage after implementation of these interfacial modifications in flexible PSCs. Further investigations on absorber/hole transport layer (HTL) interface point out the detrimental role of dopants in Spiro-OMeTAD film (widely employed HTL in the community) as recombination centers upon oxidation and light exposure.

## Graphical abstract

**Figure:**
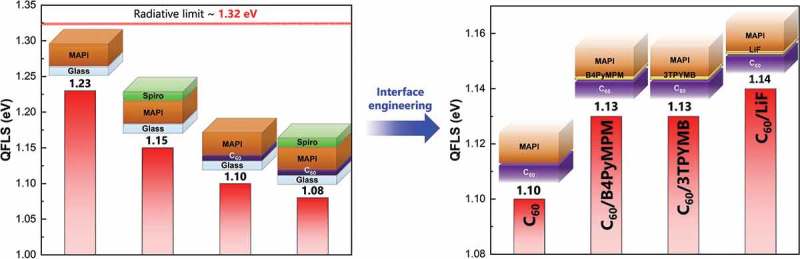

## 1. Introduction

Organic-inorganic hybrid perovskite solar cells (PSC) have achieved a certified record efficiency of 23.7%, overtaking other more mature thin-film PV technologies, like CdTe and CIGS [1,2]. One of the major reasons why PSCs have reached a widespread interest in the research community is because of their processing versatility, from fully solution- or vacuum-based processing to a hybrid combination of vacuum-solution deposition methods [3–6]. Moreover, highly efficient PSCs can be deposited via low-temperature processes [7,8], a basic requirement for the future development of lightweight and flexible modules by high-throughput roll-to-roll manufacturing [9,10].

To further improve the efficiency of PSCs, it is necessary to approach the thermodynamic efficiency limit by suppressing non-radiative recombination losses [11]. PSCs comprise heterojunction interfaces, which are characterized by interfacial defects and band bending, all of which affect charge transfer and recombination across interfaces. Moreover, during annealing, halide and methylammonium ions can be lost from the crystal, leaving under-coordinated Pb atoms (defect sites) at the surface [12]. Notably, the interfaces between the absorber and the charge selective materials, electron, and hole transport layers (ETLs and HTLs), play a key role in extracting photo-generated carriers. In order to reduce non-radiative recombination, methods for interfacial defect passivation need to be implemented. Successful approaches rely on interfacial passivation via 2D perovskites [13], functional organic molecules [12] and thin insulating layers [14]. In particular, the use of Lewis base or acid molecules can suppress positively charged trap sites (under-coordinated Pb ions or halide vacancies) or negatively charged defects (under-coordinated halide), respectively [15].

Fullerene (C_60_)-based materials represent an interesting candidate for efficient low-temperature deposited ETL, well-known as passivation against hysteresis in PSCs [5,16]. According to literature, the main strategies to suppress interfacial recombination via engineering of fullerene-based molecules are based on solution processing [17,18]. Nevertheless, the community would also benefit from interface engineering approaches for vacuum deposited C_60_ in *n-i-p* configurations (where the perovskite absorber is directly deposited onto the ETL), considering the potential large-area scalability of vacuum-based methods.

The assessment of interface quality between the perovskite absorber and charge extraction layers is not trivial. Recent works have demonstrated how to disentangle non-radiative recombination losses happening in the bulk perovskite and at the interfaces via absolute photoluminescence (PL) intensity, which is a direct measure of the quasi-Fermi level splitting (QFLS) [19–22]. A few studies have reported on how different interlayers can affect the QFLS, but mostly for *p-i-n* configurations (where the ETL is processed on top of the perovskite absorber) where the use of very thin vacuum deposited interlayers is not restricted by the solution-based processing of perovskite absorber [19,22,23].

Here we investigate voltage losses (due to non-radiative recombination) for a traditional low-temperature-processed *n-i-p* structure based on vacuum deposited C_60_ as ETL. After having identified the formation of the ETL/perovskite interface as a significant source of non-radiative recombination losses, we screened several possible interlayers to suppress interfacial recombination. By using a two-step hybrid vacuum-solution deposition for the perovskite absorber we were able to apply thin vacuum deposited interlayers at ETL/absorber interface avoiding any possible damage from solvent processing directly on the interlayer. In particular, we investigated how lithium fluoride (LiF), 2,9-Dimethyl-4,7-diphenyl-1,10-phenanthroline (BCP), 4,6-Bis(3,5-di(pyridin-4-yl)phenyl)-2-methylpyrimidine (B4PyMPM) and Tris(2,4,6-trimethyl-3-(pyridin-3-yl)phenyl)borane (3TPYMB) affect the QFLS of perovskite when deposited at ETL/absorber interface. We observed an increase of ~30–40 meV in QFLS that is comparable to the improvements in the open-circuit voltage (*V*_oc_) observed when these interlayers are implemented in flexible PSCs. Further investigations have shown that, the initially underestimated absorber/HTL interface also represents a significant source of non-radiative recombination losses, potentially due to detrimental chemical modifications at the perovskite/HTL interface as a consequence of oxidation and light exposure of the doped HTL material.

## 2. Experimental section

### 2.1. Materials

InZnO (IZO) target was bought from JX Nippon Mining & Metals (99.9%). Spiro-OMeTAD was bought from Merck. Polyethylenimine, 80%-ethoxylated solution, 35–40 wt.% in H_2_O, 4-tertbutylpyridine (TBP) lithium fluoride (LiF) powder, and lithium-bis(trifluoromethanesulfonyl)imide (Li-TFSI) were bought from Sigma-Aldrich. CH_3_NH_3_I (powder, ITEM# MS101000) and PbI_2_ (ultra-dry, 99.999%, metals basis) were purchased from Dyesol (Australia) and Alfa Aesar, respectively. Fullerene carbon 60 powder (C_60_) was bought from SES Research (purity > 99.5%). Bathocuproine (BCP) and 4,6-Bis(3,5-di(pyridin-4-yl)phenyl)-2-methylpyrimidine (B4PyMPM) were purchased from Angstrom Engineering Inc. and Tris(2,4,6-trimethyl-3-(pyridin-3-yl)phenyl)borane (3TPYMB) was bought from Luminescence Technology Corp. (Lumtec). Tin(IV) oxide colloidal dispersion was purchased from Alfa Aesar. For *a*-TiO_2_ layer by ALD: tetrakis(dimethylamino)titanium(IV) (TDMAT) 5N was bought from Merck. All chemicals were used as received without any further treatment for purification.

### 2.2. Device fabrication

PSCs were grown on flexible foil which is used as a moisture barrier front sheet for encapsulation in flexible CIGS modules. 5 cm × 5 cm-size flexible substrates were washed by hand followed by ultrasonic soap and water baths. The substrates were dried in vacuum for one week and cut into four quarters (2.5 cm × 2.5 cm). Prior to further processing, ~200 nm of compact IZO layer was deposited at room temperature by pulsed-DC sputtering from a ceramic In_0.89_Zn_0.11_O target, at 600 W. The sheet resistance of as-deposited film on glass is 18 Ω□ measured by four-probe method. Then, IZO layer was treated for 5 min under O_2_ plasma-treatment for all the samples. PEIE interlayer (0.1% w/w) in deionized (DI) water was deposited onto IZO layer by spin coating (5000 rpm, 5000 rpm s^−1^ for 60 s) and subsequent annealing at 100°C for 10 min. Then, ~5 nm of C_60_ was thermally evaporated in the N_2_ filled glovebox at a rate ~0.2 Å s^−1^. In case of interfacial modification, ~1 nm of BCP, B4PyMPM, 3TPYMB, and LiF were thermally evaporated in the N_2_ filled glovebox at a rate ~0.1–0.2 Å s^−1^. When using SnO_2_ as ETL, the deposition recipe followed exactly what reported elsewhere [7]. When using TiO_2_ as ETL: *a*-TiO_2_ layer was deposited by ALD at a substrate temperature of 100°C from TDMAT and H_2_O with a Fiji G2 system (Ultratech). Ar was used as carrier gas at a base pressure of 10 Pa. The PbI_2_ film was thermally evaporated at a deposition pressure of 2–6 ×10^−6^ Pa. The deposition rate was controlled within 1.2–1.6 Å s^−1^, monitored by a quartz crystal microbalance. The thickness of PbI_2_ is ~160 nm. After the PbI_2_ deposition, the samples were subsequently transferred into a N_2_ filled glovebox for further processing. The perovskite layer was formed by spin coating of CH_3_NH_3_I in 2-propanol at a concentration of 55 mg mL^−1^. The solution was first spread to cover the whole substrate, and wait for 5 s before starting the rotation (4000 rpm, 4000 rpm s^−1^ for 40 s). The as-prepared films were annealed at 100°C for 30 min on a hotplate under a fume hood, outside of the glovebox. The measured relative humidity was between 20% and 30% (temperature ~22°C) close to the hotplate. After annealing, the samples were cooled down to room temperature and 100 μL of a Spiro-OMeTAD solution (78.2 mg 2,2ʹ,7,7ʹ-tetrakis-(N,N’-di-p-methoxyphenylamine)-9,9ʹ-spirobifluorene (Spiro-OMeTAD)), 33 μL lithium-bis(trifluoromethanesulfonyl)imide (Li-TFSI) solution (170 mg Li-TFSI in 1 mL acetonitrile, Sigma-Aldrich), and 8.2 μL 4-tertbutylpyridine (TBP) all dissolved in 1 mL of chlorobenzene (Sigma-Aldrich) was spin-coated on top of perovskite at 2500 rpm, 2500 rpm s^−1^ for 45 s. The devices were finished by evaporating 50 nm Au through a metal mask under high vacuum (<3×10^−4^ Pa). The solar cell active area is equal to 0.15 cm^2^.

### 2.3. Characterization

Absolute PL: Excitation for the PL imaging measurements was performed with a 445 nm CW laser through an optical fiber into an integrating sphere (Ulbricht sphere). The intensity of the laser was adjusted to a 1 sun equivalent intensity by illuminating a 1 cm^2^-size perovskite solar cell under short-circuit and matching the current density to the short-circuit current density (*J*_sc_) under the solar simulator. A second optical fiber was used from the output of the integrating sphere to a spectrometer equipped with a silicon CCD camera. The system was calibrated by using a calibrated halogen lamp with specified spectral irradiance, which was shone into the integrating sphere. A spectral correction factor was established to match the spectral output of the detector to the calibrated spectral irradiance of the lamp. The spectral photon density was obtained from the detector-corrected signal (spectral irradiance) by dividing with the photon energy, and the photon numbers of the excitation and emission obtained from numerical integration using Matlab. In a last step, three fluorescent test samples with high-specified absolute external PL quantum yield (PLQY; ~70%) supplied from Hamamatsu Photonics were measured and the specified value could be accurately reproduced within a small relative error of less than 5%. The PL of the samples was recorded after an exposure of ~10 s to the laser light after mounting the sample. Therefore, the PLQY is obtained on timescales relevant to the *V*_oc_ measurements of the PSCs. All absolute PL measurements were performed on films with the same ETL, HTL and perovskite thicknesses as used in the PSCs. The absorption of the samples was considered in the PLQY calculation and was ~84% for cells illuminated through the perovskite, and ~93% through the glass substrate. The QFLS was measured by illuminating the films through the perovskite or glass substrate in order to avoid parasitic absorption in the investigated charge transport layers. The illuminated area is 1 cm^2^. The global systematic error of the QFLS is ~25 meV, or an uncertainty of ± 12.5 meV for a given QFLS, calculated considering upper and lower limits for each parameter that define the QFLS (temperature ± 2°C, $J_{G}$ ± 2 mA/cm^2^, PLQY ± 25% relative).

*J-V* characteristics and external quantum efficiency (EQE): The current density-voltage characteristics of PSCs are measured under standard-simulated AM1.5G illumination using a Keithley 2400 source meter. The illumination intensity is calibrated to 1000 W m^−2^ using a certified single crystalline silicon solar cell. The *J-V* measurement is performed in both forward (form −0.1 V to 1.4 V) and backward (from 1.4 V to −0.1 V) direction separately without any pretreatment (e.g. light soaking, holding at forward bias for certain time etc.). The scan rate and delay time are 0.3 V s^−1^ and 10 ms, respectively. The external quantum efficiency is measured with a lock-in amplifier. The probing beam is generated by a chopped white source (900 W, halogen lamp, 260 Hz) and a dual grating monochromator. The beam size is adjusted to ensure that the illumination area is fully inside the cell active area. A certified single crystalline silicon solar cell is used as the reference cell. White light bias is applied during the measurement with ~0.1 sun intensity. The steady-state efficiency as a function of time is recorded using a maximum power point tracker, which adjusts the applied voltage in order to reach the maximum power point (perturb and observe algorithm). The starting voltage is set to be 0.1 V.

## 3. Results and discussion

The low-temperature deposited PSC structure comprises a CH_3_NH_3_PbI_3_ (MAPI) absorber which is sandwiched between vacuum deposited C_60_, as ETL, and spin coated Spiro-OMeTAD (Spiro), as HTL [10]. The perovskite is deposited via a two-step hybrid vacuum-solution deposition method, where PbI_2_ is thermally evaporated and then converted to MAPI phase by CH_3_NH_3_I spin coating and further annealing [5]. This process enables to deposit perovskite absorbers on solvent-sensitive layers like C_60_, avoiding the use of dimethyl sulfoxide/dimethylformamide-mixed solvents (commonly employed for solution-processed perovskites [3]). Figure S1(a) shows a representative *J-V* characteristic of the PSC grown onto the flexible substrate, which is the front sheet used to encapsulate flexible Cu(In,Ga)Se_2_ (CIGS) modules, as we already reported elsewhere [9,10,24]. The device displays a short-circuit current density (*J*_sc_) of 19.1 (19.1) mA/cm^2^, an open-circuit voltage (*V*_oc_) of 1.05 (1.04) V and a fill factor (FF) of 71.9 (72.5) % under forward (backward) measurement. The inset displays the maximum power point (MPP) tracking under 1 sun continuous illumination. A steady-state power conversion efficiency of 14.2% is obtained. Figure S1(b) shows a statistics of *V*_oc_ values for >20 devices. An average *V*_oc_ value of ~1.05 V is observed for this low-temperature PSC structure.

An efficient method to enhance the *J*_sc_ values has already been demonstrated in our previous work [10] combined with optical loss analyses to define future pathways for efficiency improvements. The limited *V*_oc_ values indicate substantial room for improvement. This work is targeted on the analysis of the voltage losses and propose methods to reduce non-radiative recombination losses for a typical low-temperature deposited *n-i-p* PSC structure. Firstly, it is necessary to assess if the *V*_oc_ is limited by recombination losses happening in the bulk of perovskite or at the interfaces with other layers. Stolterfoht et al. have developed a simple and useful method to disentangle bulk from interfacial and contact-mediated non-radiative recombination losses through measurement of the PLQY that is emitted from individual perovskite/transport layer films of the cell [22]. The measurement of the PLQY allows to quantify the QFLS or free energy of the electron and hole pairs created in the films upon a 1 sun equivalent illumination.

(1) $$QFLS=kTln\left(PLQY\frac{J_{G}}{J_{0,rad}}\right)$$

where k is the Boltzmann’s constant, T is the absolute temperature (26°C, i.e. kT= 25.8 meV), $J_{G}$ and $J_{0,rad}$ are the generated current densities under illumination and in the dark, respectively. $J_{G}$ (~190 A/m^2^) and $J_{0,rad}$ (~1 × 10^−20^ A/m^2^) are obtained from integration of the EQE with the solar and 300 K-black body spectrum, respectively (Figure S2). Equation (1) has been first proposed by Ross [25] and it has been widely employed in the inorganic solar cell community [26]. It is a direct result of the equation for the radiative recombination current density according to Shockley-Queisser [27]:

(2) $$J_{rad}=J_{0,rad}\cdot e^{QFLS/kT}$$

Two conditions need to be fulfilled:

1. $J_{rad}$ and $J_{0,rad}$ must have the same spectral dependence, which means that recombination goes through the same channels regardless of the QFLS;
2. The emission must come from free charges, as only free charges (and not strongly bound excitons) create a QFLS.

The calculation of $J_{0,rad}$ from the EQE considers negligible transport losses. The PLQY is defined by the ratio of emitted and absorbed photon fluxes. Considering the condition that recombination originates from free charges, the PLQY can be expressed as a ratio of $J_{rad}/J_{G}$, which then will result in Equation (1).

**Figure 1.**
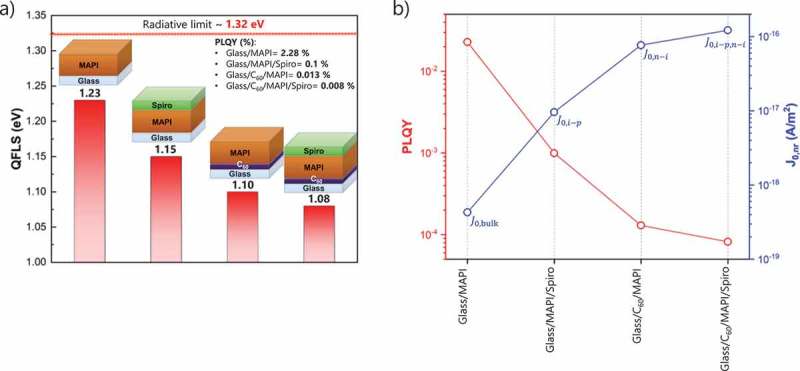
(a) Calculated QFLS of the different studied heterojunctions (glass/MAPI, glass/MAPI/Spiro, glass/C_60_/MAPI and glass/C_60_/MAPI/Spiro) based on Equation 1 using absolute PL measurements. (b) PLQY and individual $J_{0,nr}$ contributions (calculated from Equation 3) for the different heterojunctions.

First, we used this method to assess the QFLS of the bare MAPI absorber deposited by two-step hybrid approach on glass, and to study how the QFLS changes when MAPI is deposited on C_60_ due to the formation of a new interface. For the bare perovskite deposited on glass, we observed a QFLS of ~1.23 eV that is only ~90 meV below the radiative *V*_oc_ limit (~1.32 eV) where the PLQY is equal to 1 (Figure 1(a)). As pointed out previously [22], this value could be limited by the glass/perovskite interface which has been observed to be worse compared to a fused silica/perovskite interface in case of a triple cation perovskite. Other studies have also highlighted the major importance of the top surface, i.e. by passivating MAPI with tri-n-octylphosphine oxide (TOPO), Braly and Hillhouse [28] achieved a very high QFLS of 1.28 eV with an external PLQY of 20% very close to the radiative limit. Hence, we conclude that the QFLS potential of the perovskite bulk may be even higher than what we obtained if the surfaces are properly passivated. Regardless of that, when MAPI is deposited on thermally evaporated C_60_, the QFLS drops to ~1.10 eV, indicative of strong non-radiative recombination losses (Figure 1(a)) arising from the formation of the new C_60_/MAPI interface. Figure S3 shows the corresponding PL spectra. In order to understand the recombination losses at the interface, we note that PLQY can be defined as [22]:

(3) $$PLQY=\frac{J_{0,rad}}{J_{0,rad}+J_{0,nr}}=\frac{J_{0,rad}}{J_{0,rad}+J_{0,bulk}+J_{0,i-p}+J_{0,n-i}+\;\ldots \;}$$

where the non-radiative dark saturation current ($J_{0,nr}$) is given by the sum of all non-radiative recombination pathways ($J_{0,bulk}$ = non-radiative recombination in the bulk,$\;J_{0,i-p}$ = non-radiative recombination at MAPI/Spiro interface, $J_{0,n-i}$ = non-radiative recombination at C_60_/MAPI interface). As shown in Figure 1(a), the PLQY decreases from ~2.28 to ~0.013%. This means that $J_{0,nr}$ increases more than 2 orders of magnitude from 4.3×10^−19^ ($J_{0,bulk}$) to ~7.7×10^−17^ A/m^2^ ($J_{0,bulk}$+$J_{0,n-i}$) due to the presence of additional interfacial recombination (Figure 1(b)). When the MAPI absorber is sandwiched between C_60_ and Spiro, the QFLS decreases further with respect to C_60_/perovskite stack to a value ~1.08 eV ($J_{0,n-i}$+$J_{0,i-p}$= 1.2×10^−16^ A/m^2^), which is comparable to the average *V*_oc_ obtained for flexible PSCs. We note that we cannot rule out slightly different perovskite crystallizations when the perovskite is deposited on a glass or C_60_ substrate, which could potentially affect the QFLS of the bulk. However, the growth of thermally evaporated PbI_2_ shows a similar compact morphology when deposited onto amorphous substrates like glass or C_60_ [29], suggesting a similar perovskite phase crystallization. Moreover, a significant increase in non-radiative recombination losses is also observed when C_60_ is deposited on top of the perovskite absorber [19,22]. For these reasons, we believe that the drop in QFLS is mainly dictated by additional non-radiative recombination losses at C_60_/MAPI interface.

**Figure 2.**
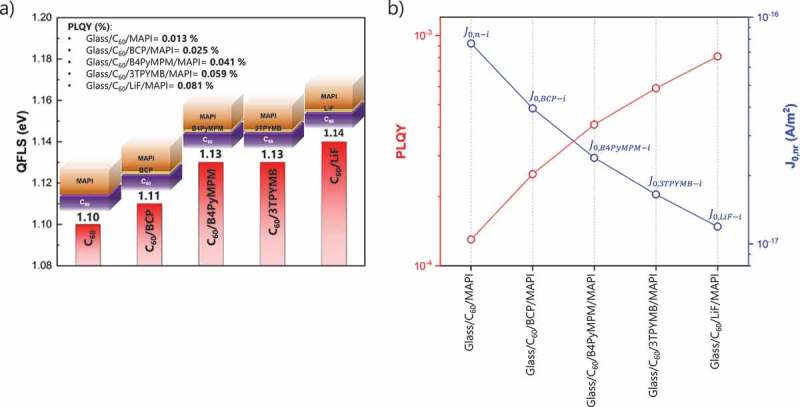
(a) Calculated QFLS for the different interfacial modifications (BCP, B4PyMPM, 3TPYMB and LiF) with respect to glass/C_60_/MAPI heterojunction. (b) PLQY and individual $J_{0,nr}$ contributions for the different heterojunctions.

**Figure 3.**
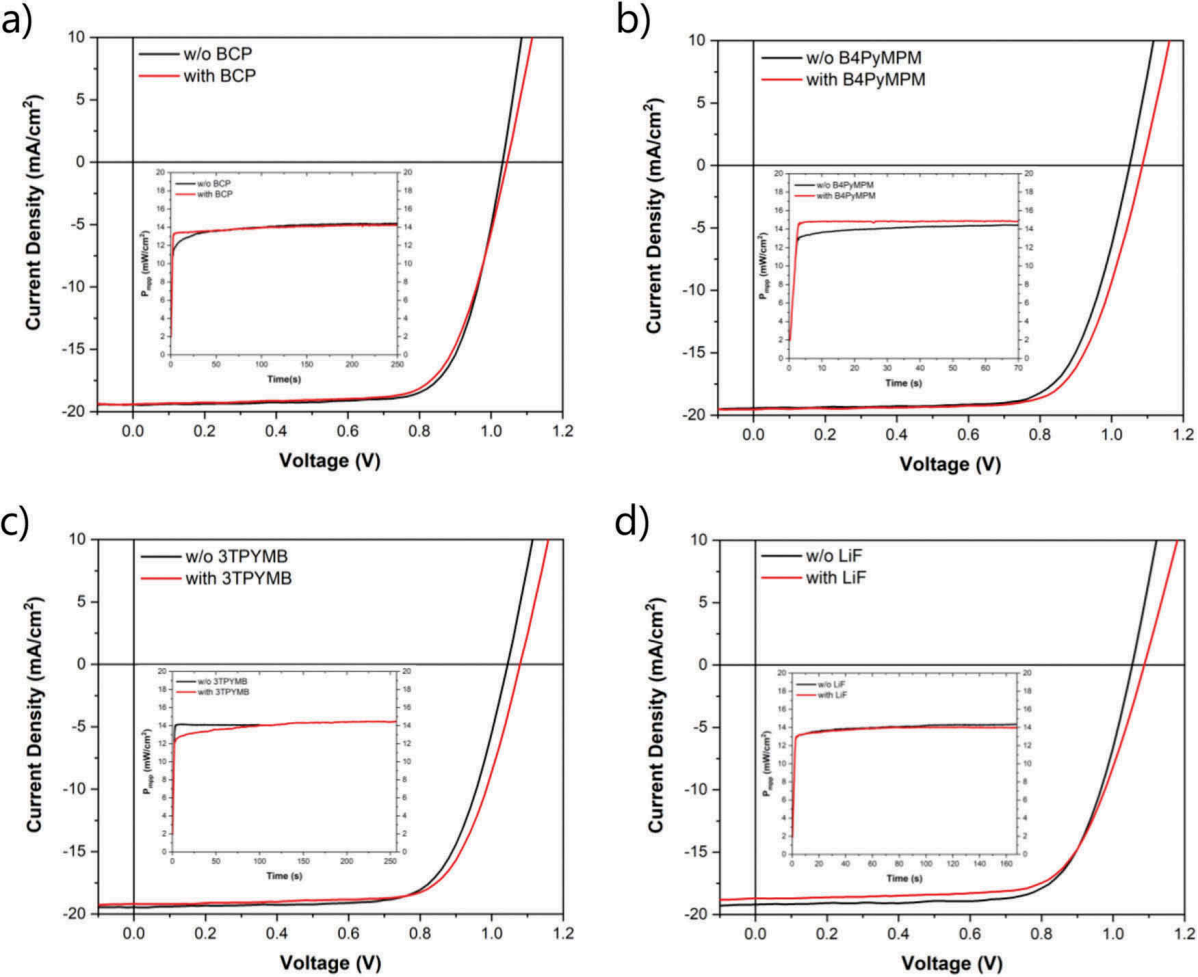
J-V characteristics of flexible PSCs with and without (a) BCP, (b) B4PyMPM, (c) 3TPYMB and (d) LiF interfacial modifications. The inset shows the comparison of power outputs at MPP under 1 sun continuous illumination with and without interlayers.

In order to reduce the non-radiative recombination, we investigated and compared different materials (LiF and pyridine-derivatives: BCP, B4PyMPM, and 3TPYMB) as ETL/MAPI interfacial modifications (thickness ~1 nm). Figure 2(a) shows how the QFLS changes upon deposition of the different interlayers. When BCP is deposited onto C_60_, the QFLS remains unchanged (negligible improvement), ~1.11 eV. When B4PyMPM and 3TPYMB are applied an improvement of about 30 meV is observed in the QFLS (from ~1.10 to ~1.13 eV) with respect to the bare C_60_ as ETL. Also, LiF increases the QFLS, by 40 meV with respect to the bare C_60_ (from ~1.10 to ~1.14 eV), a comparable enhancement to what is observed elsewhere [19]. The corresponding PL spectra are displayed in Figure S4. Figure 2(b) shows the comparison among PLQYs and the different $J_{0,nr}$ contributions. A reduction in non-radiative recombination losses with respect to C_60_/MAPI interface is observed by ~2-fold with BCP, ~3-fold with B4PyMPM, ~4.5-fold with 3TPYMB and ~6-fold with LiF. According to literature [12,30–32], the reduction of non-radiative interfacial recombination can be related to the electronic passivation of positively charged defects (like halide vacancies) when Lewis base molecules are applied (like pyridine-derivatives) and to formation of interfacial dipoles which could positively affect the energy level alignment, improving the quality of electrical contacts. We then implemented these vacuum deposited interlayers in the *n-i-p* flexible PSC structure. Figure 3 shows the representative *J-V* characteristics for the different batches and Figures S5-8 display the statistics. We want to underline that for every interfacial modification investigated, a reference sample (without interlayer) was prepared in the same batch. Therefore, the PV performances of the devices with interfacial modifications will only be compared to the corresponding references, in this way we are sure that all the layers in the compared samples are deposited and processed in the same run, from the same precursors, under the same glovebox conditions and same relative humidity conditions during perovskite annealing. Figure 3(a) shows the representative *J-V* characteristics for the flexible PSCs with and without BCP interlayer. Without BCP, we obtain a *V*_oc_ of 1.03 V, a *J*_sc_ of 19.5 mA/cm^2^ and a FF of 74.3%. At MPP under 1 sun continuous illumination we achieve a steady-state efficiency of 14.4%. When BCP is deposited between C_60_ and MAPI, a slightly better *V*_oc_ of 1.05 V is demonstrated, *J*_sc_ of 19.3 mA/cm^2^ and a decreased FF of 71.9%. An efficiency of 14.2% is obtained. As expected from QFLS evaluation (Figure 2(a)), we did not observe a notable improvement in *V*_oc_ by applying BCP interlayer. Figure 3(b) shows the flexible devices with and without B4PyMPM interfacial modification. We observe a *V*_oc_ of 1.09 (1.05) V, *J*_sc_ of 19.5 (19.5) mA/cm^2^, a FF of 71.7 (71.7) % and an efficiency at MPP of 14.9 (14.5) % with (without) B4PyMPM interlayer.

Figure 3(c) shows the representative *J-V* characteristics for the flexible PSCs with and without 3TPYMB interlayer. For the reference, we observed a *V*_oc_ of 1.05 V, *J*_sc_ of 19.5 mA/cm^2^, FF of 71.1% and a steady-state efficiency of 14.1%. When 3TPYMB is applied the *V*_oc_ is improved to 1.08 V, while the *J*_sc_ and FF values are comparable to the reference: 19.2 mA/cm^2^ and 71.5%, respectively. An efficiency at MPP under 1 sun continuous illumination of 14.5% is demonstrated. Eventually, the *J-V* curves for the devices with and without LiF interfacial modification are shown in Figure 3(d). We achieved a *V*_oc_ of 1.09 (1.05) V, *J*_sc_ of 18.7 (19.3) mA/cm^2^, a FF of 69.3 (71.2) % and an efficiency at MPP of 14.0 (14.3) % with (without) LiF interfacial modification. Figure S9 displays the corresponding EQE measurements of the reported solar cells. Table 1 shows a summary of the PV parameters of the devices presented in Figure 3.

**Table 1.** PV parameters from J-V characteristics shown in Figure 3.

Solar cell	*V*_oc_ (V)	*J*_sc_ (mA/cm^2^)	FF (%)	*η*_MPP_ (%)
w/o BCP	1.03	19.5	74.3	14.4
with BCP	1.05	19.3	71.9	14.2
w/o B4PyMPM	1.05	19.5	71.7	14.5
with B4PyMPM	1.09	19.5	71.7	14.9
w/o 3TPYMB	1.05	19.5	71.1	14.1
with 3TPYMB	1.08	19.2	71.5	14.5
w/o LiF	1.05	19.3	71.2	14.3
with LiF	1.09	18.7	69.3	14.0

By comparing these results with the previous QFLS evaluations, we observe a good matching between QFLS and *V*_oc_ improvements. From QFLS studies an almost negligible variation in attainable *V*_oc_ was observed when BCP is applied, while when B4PyMPM, 3TPYMB, and LiF are applied an improvement of ~30–40 meV was measured, which is very similar to the *V*_oc_ improvements observed in solar cells. However, as shown in Figures S5-S8, the devices experience a general reduction in FF. Further investigations are needed in these regards, but we can propose that this reduction is due to an increased contact resistance (*R*_s_, series resistance) as can be observed by the representative *J-V* characteristics (Figure 3) and by the corresponding *R*_s_ values extracted from the corresponding dark *J-V* curves (Figure S10). As shown in Table S1, a general increase in *R*_s_ is observed when the different interlayers are applied. Considering the large optical gap of these interlayers, we expect that they detrimentally affect the charge transfer to the transport layers due to possibly unfavorable energy band alignment.

**Figure 4.**
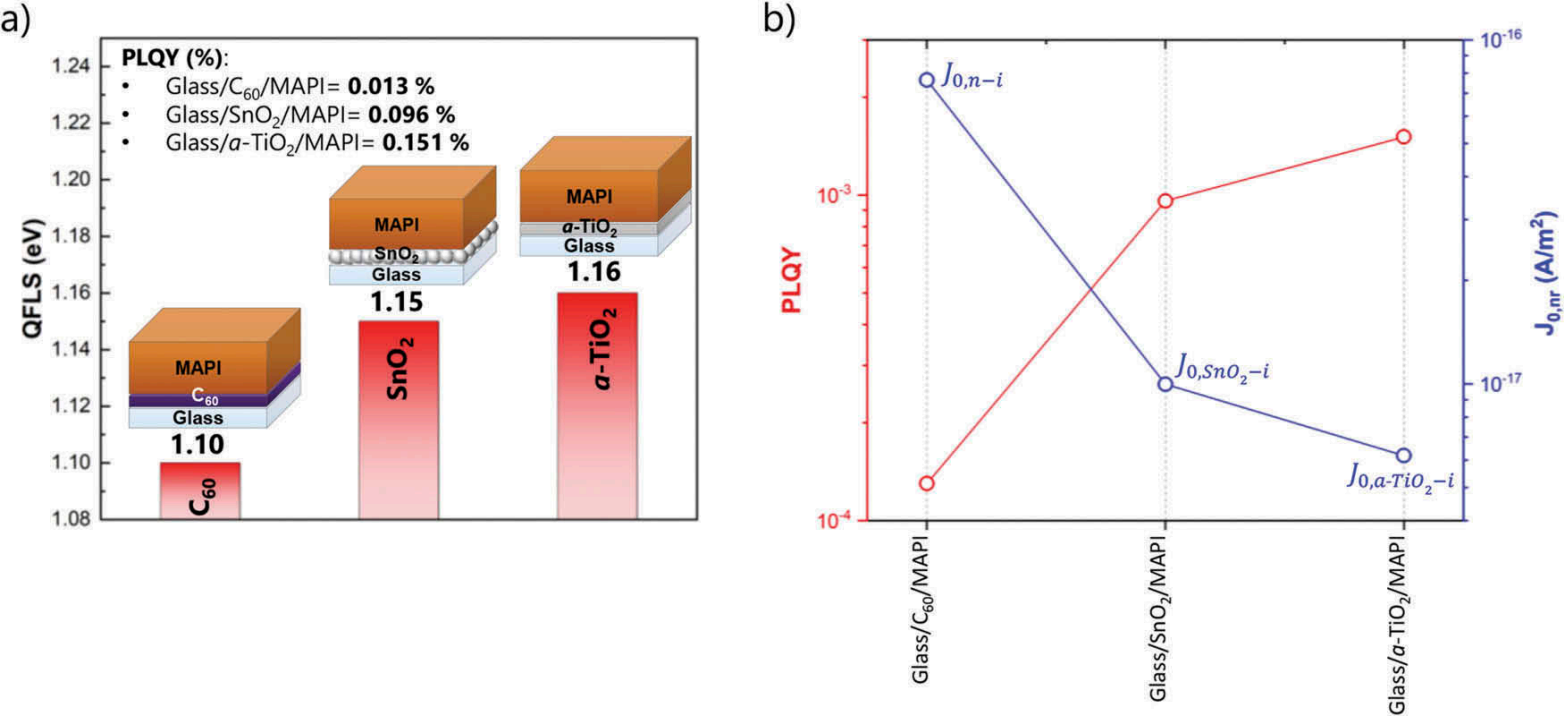
(a) Calculated QFLS of the different-investigated heterojunctions (glass/C_60_/MAPI, glass/SnO_2_/MAPI and glass/a-TiO_2_/MAPI/Spiro). (b) PLQY and individual $J_{0,nr}$ contributions for the different ETL/MAPI interfaces.

**Figure 5.**
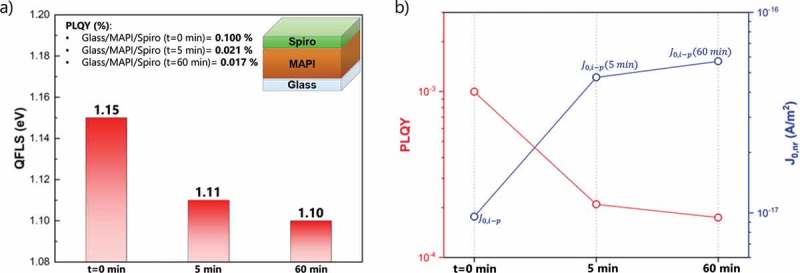
(a) Calculated QFLS for glass/MAPI/Spiro heterojunction at different times under ambient atmosphere exposure. (b) PLQY and individual $J_{0,nr}$ contributions as a function of time.

Another possibility to reduce *V*_oc_ losses, would be to directly substitute C_60_ with other efficient low-temperature-deposited ETLs, like SnO_2_ or TiO_2_. We investigated how spin coated SnO_2_ and ALD-deposited amorphous TiO_2_ (*a*-TiO_2_) would affect the QFLS of perovskite. Figure 4(a,b) show the corresponding QFLS, PLQY and $J_{0,nr}$ (PL spectra in Figure S11). A significant improvement from 1.10 eV to 1.15 and 1.16 eV is observed by substituting C_60_ with SnO_2_ and *a*-TiO_2_, respectively (Figure 4(a)). With respect to C_60_/MAPI interface, an ~eightfold reduction of non-radiative recombination is observed when SnO_2_ is used (Figure 4(b)). An even further reduction is achieved with *a*-TiO_2_ as ETL (~12-fold reduction) (Figure 4(b)). However, when implemented in solar cells, the measured *J-V* characteristics and performances are very poor, showing rather strong hysteresis and very low and unstable efficiency at MPP under 1 sun continuous illumination (Figures S12). We observed similar poor PV performances with other low-temperature oxide-based ETLs [5]. We believe that appropriate surface modification strategies for these oxide-based ETLs would be necessary to have well-working devices (this subject is under investigation in a separate work). This shows that a charge transport layer that demonstrates high QFLS is necessary but not sufficient for producing efficient PV devices, also resistive losses and charge extraction properties have to be considered. However, the low *V*_oc_ values of the devices with metal oxide transport layers may also indicate that the top interface, which was initially thought to be not limiting, can be as detrimental as the C_60_/MAPI junction. By analyzing the interface between MAPI and doped Spiro more carefully, we observed that the QFLS tends to decrease with time (Figure 5(a,b)). The corresponding PL spectra are displayed in Figure S13. When MAPI is coated with Spiro, the QFLS is initially ~1.15 eV (i.e. much better than the QFLS of the C_60_/MAPI film ~1.10 eV). However, by keeping the sample under the 1 sun intensity laser and in the ambient atmosphere the QFLS decreases to ~1.11 eV after 5 min (comparable times used during MPP tracking) which further drops to ~1.10 eV after 1 h. Notably, this behavior is not observed for the other films which exhibit a stable QFLS. This decrease is possibly due to the progressive oxidation of the HTL doped with Li-TFSI. Unlike chemical oxidants, Li-TFSI does not oxidize Spiro directly but it supports the oxidative reaction with oxygen in the presence of light excitation [33]. As also pointed out by Correa-Baena et al. [34], the environmental oxygen affects the recombination dynamics, making dopants act as recombination centers at the perovskite/HTL interface. Possible solutions may rely on the use of different HTLs or dopants for Spiro, as recently reported [35,36]. Overall, these observations suggest that both interfaces (at the bottom and at the top) pose a substantial limitation on the *V*_oc_ for our MAPI solar cells.

## 4. Conclusions

We have performed a voltage loss analysis for a typical low-temperature *n-i-p* PSC structure based on vacuum deposited C_60_ as ETL. By using absolute PL measurements we identified the ETL/MAPI interface as a significant source of non-radiative recombination which causes a recombination current that is more than 2 orders of magnitude larger than the defect recombination current in the bare perovskite. Importantly, the QFLS of the *n-i-p* stack matches the *V*_oc_ of the cell closely, meaning that the interfacial recombination is the main loss mechanism in our devices. To mitigate these losses we investigated several interfacial modifications at the ETL/perovskite interface (BCP, B4PyMPM, 3TPYMB, and LiF). An improvement in QFLS of ~30–40 meV was observed with respect to C_60_/MAPI heterojunction by employing B4PyMPM, 3TPYMB, and LiF. Their further implementation in PSCs grown on flexible substrates support the conclusions from absolute PL, observing a comparable improvement in *V*_oc_ values when these interlayers are applied, confirming their role as passivation layers against non-radiative recombination. Further investigations of the initially underestimated MAPI/Spiro heterojunction show that the QFLS of MAPI coated with doped Spiro decreases under constant light exposure in the ambient atmosphere. These observations may confirm the detrimental role of dopants (Li-TFSI) in Spiro as MAPI/HTL interfacial recombination centers upon oxidation and light exposure. Through this voltage loss analysis, we lay the foundations for future advancements towards reaching the radiative limit and higher efficiency flexible *n-i-p* type PSCs using vacuum-deposited interlayers.
