# Supplementary material for: On the origin of open-circuit voltage losses in flexible n-i-p perovskite solar cells
Source: Sci Technol Adv Mater. 2019 Jun 21;20(1):786–95. doi: 10.1080/14686996.2019.1633952 (PMC6691881; doi:10.1080/14686996.2019.1633952)
Supplement: Supplemental Material [file TSTA_A_1633952_SM0895.pdf]

Supplementary Information

On the Origin of Open-Circuit Voltage Losses in Flexible *n-i-p* Perovskite Solar Cells

Stefano Pisoni[a*], Martin Stolterfoht[b], Johannes Loeckinger[a], Thierry Moser[a], Yan Jiang[a], Pietro Caprioglio[b,c], Dieter Neher[b], Stephan Buecheler[a] and Ayodhya N. Tiwari[a]

[a]*Laboratory for Thin Films and Photovoltaics, Empa-Swiss Federal Laboratories for Materials Science and Technology, Ueberlandstrasse 129, 8600 Duebendorf, Switzerland;*

[b]*Institute of Physics and Astronomy, University of Potsdam, Karl-Liebknecht-Strasse 24-25, D-14476 Potsdam-Golm, Germany;*

[c]*Young Investigator Group Perovskite Tandem Solar Cells, Helmholtz-Zentrum Berlin fuer Materialien und Energie GmbH, Kekuléstrasse 5, 12489 Berlin, Germany.*

***E-mail: stefano.pisoni@empa.ch**

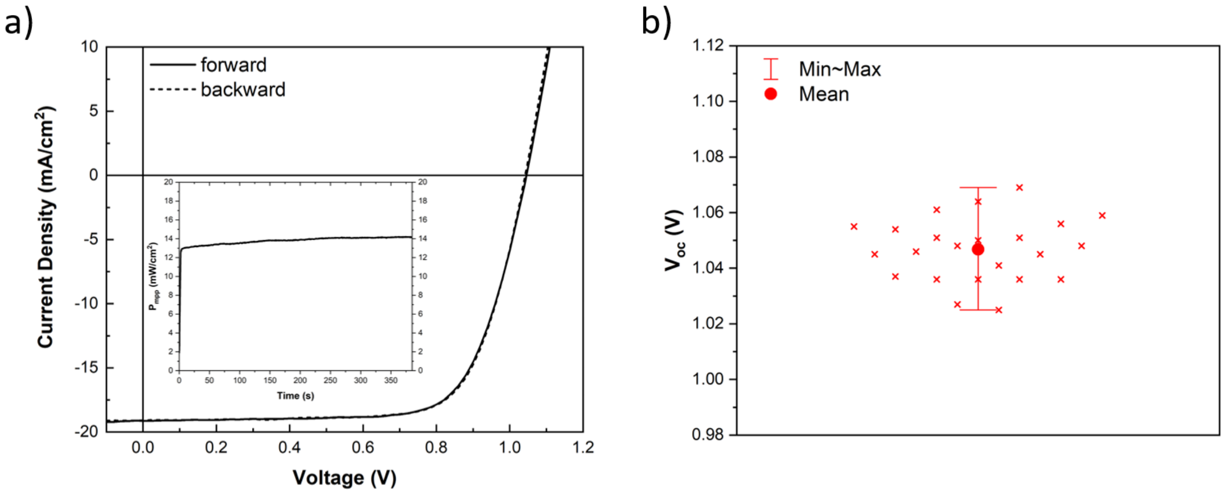

Figure S1. a) *J*-*V* characteristics of the flexible PSC under forward (-0.1 to 1.4 V) and backward (1.4 to -0.1 V) measurements. The inset shows the power output at MPP under 1 sun continuous illumination. b) Statistics of V_{oc} values for the flexible PSCs.

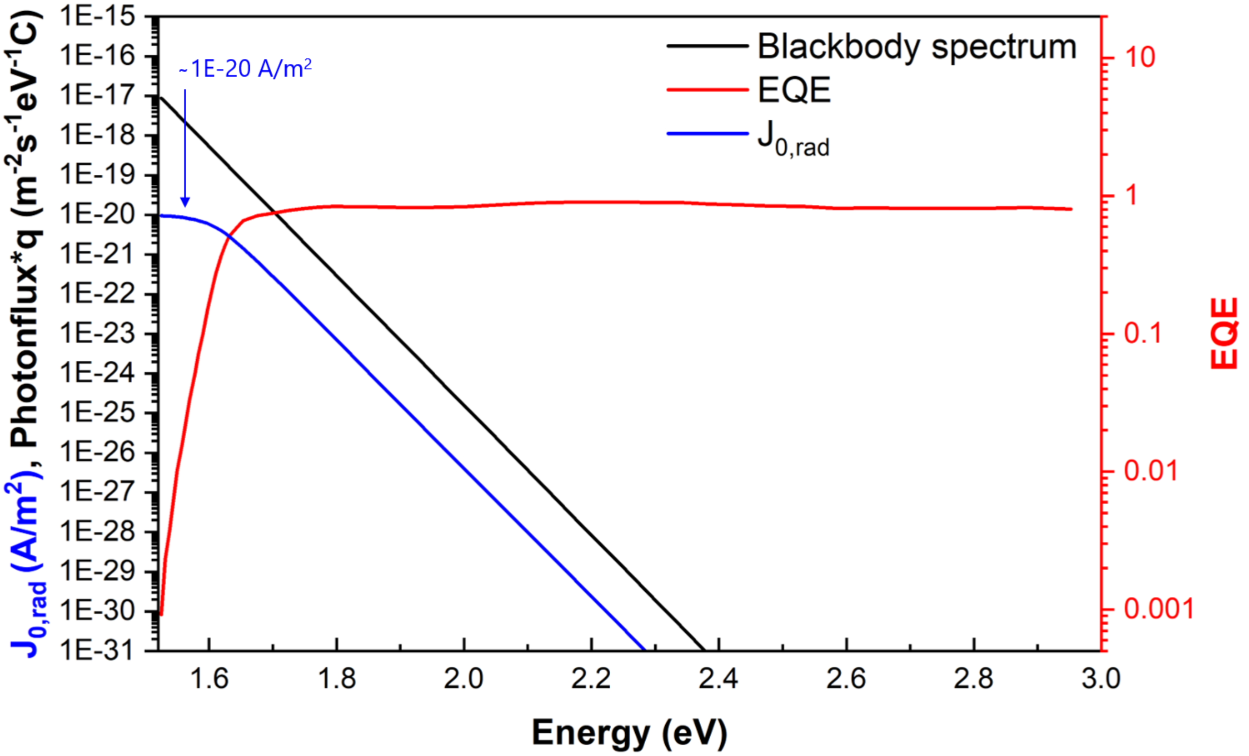

Figure S2. EQE spectrum (red curve) of our low-temperature deposited flexible PSC structure, blackbody spectrum at 300 K (black curve) and $J_{0,rad}$ calculated from the integration of blackbody spectrum*EQE. $J_{0,rad}$ is ~$1x10^{-20}$ A/m^2.

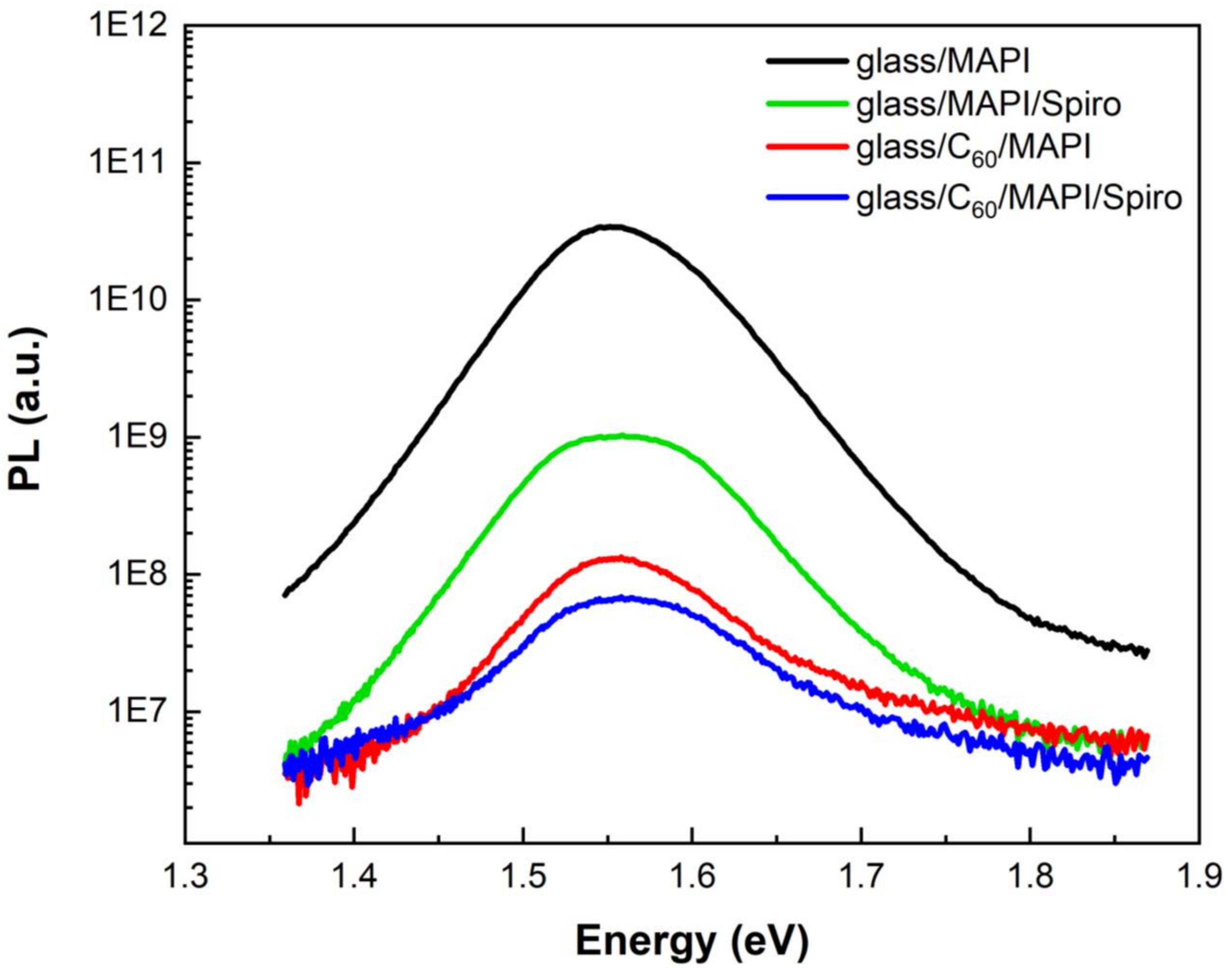

Figure S3. Comparison of PL spectra of the different heterojunctions investigated: glass/MAPI, glass/MAPI/Spiro, glass/C_{60}/MAPI and glass/C_{60}/MAPI/Spiro.

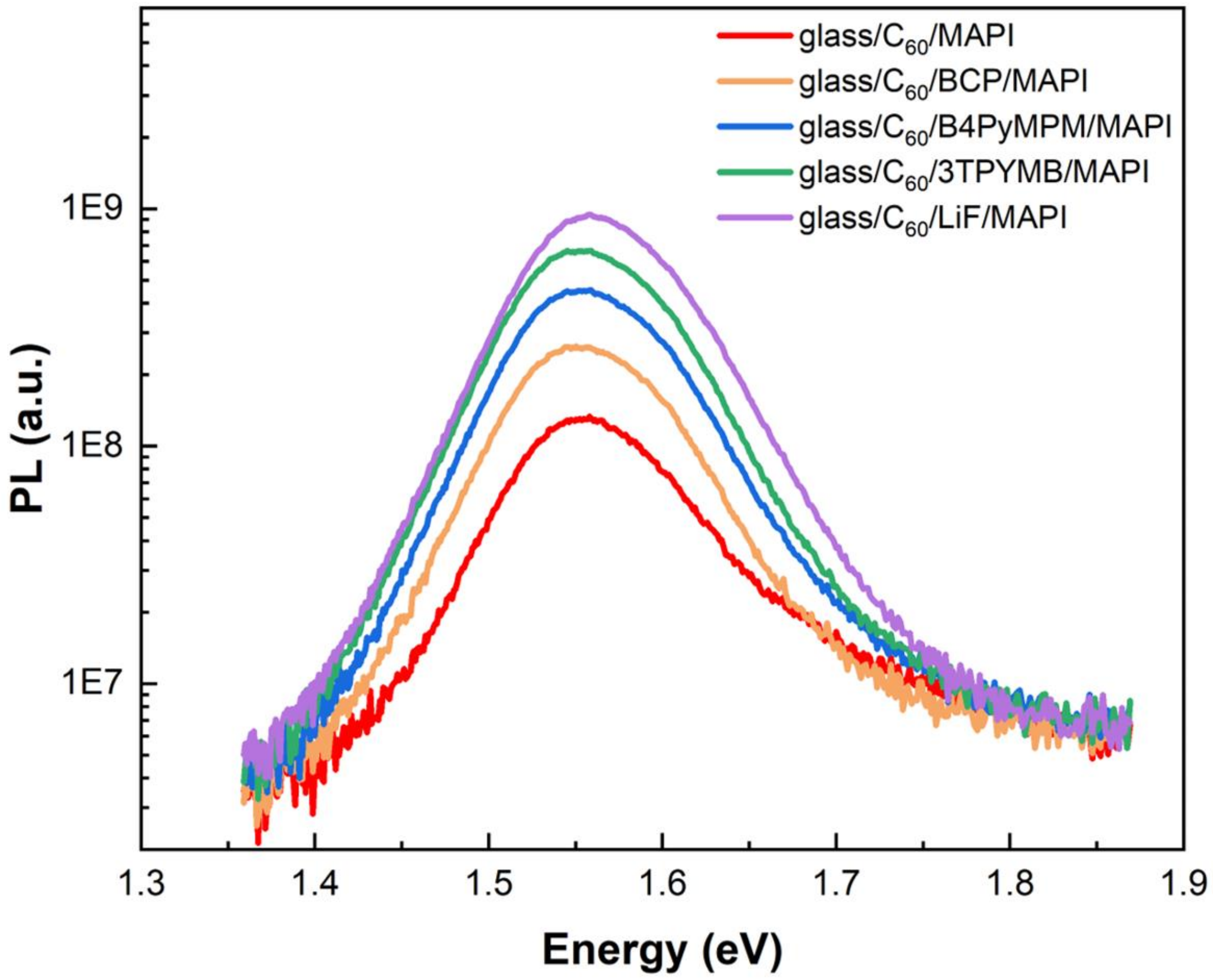

Figure S4. Comparison of PL spectra for the different interfacial modifications studied with respect to glass/C_{60}/MAPI: glass/C_{60}/MAPI, glass/C_{60}/BCP/MAPI, glass/C_{60}/B4PyMPM/MAPI, glass/C_{60}/3TPYMB/MAPI and glass/C_{60}/LiF/MAPI/Spiro.

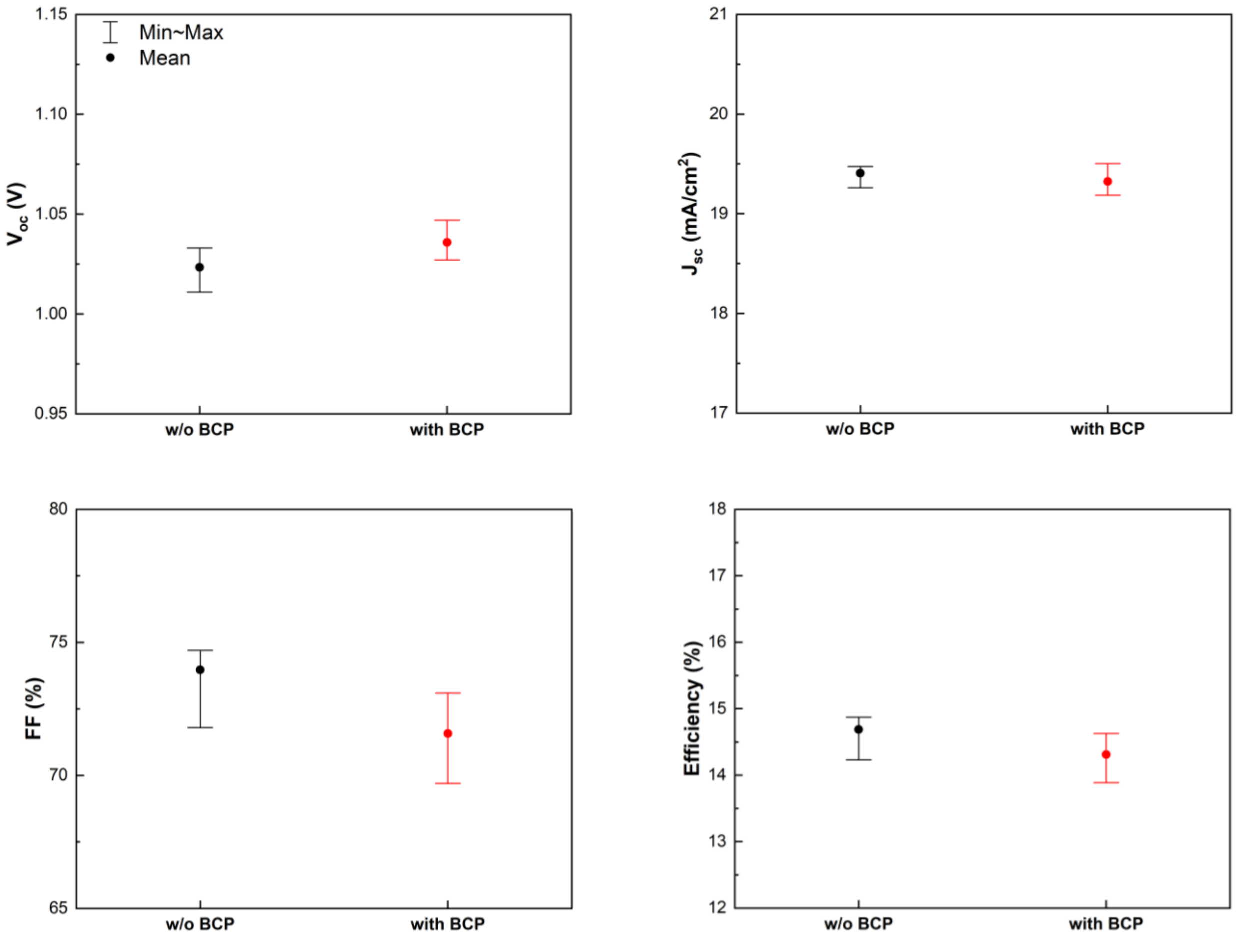

Figure S5. Comparison among PV parameters statistics of the flexible PSCs with and without BCP interfacial modification.

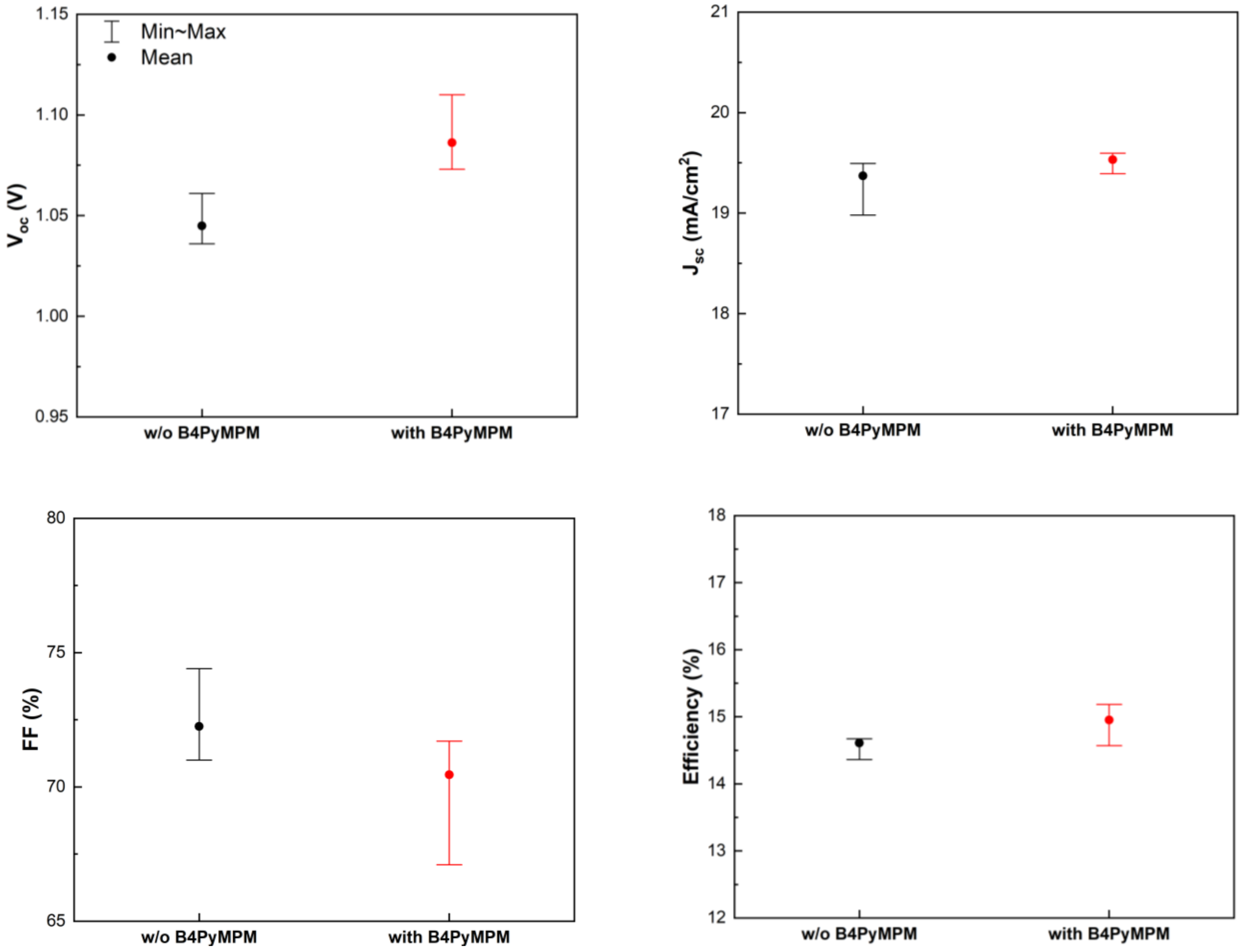

Figure S6. Comparison among PV parameters statistics of the flexible PSCs with and without B4PyMPM interlayer application.

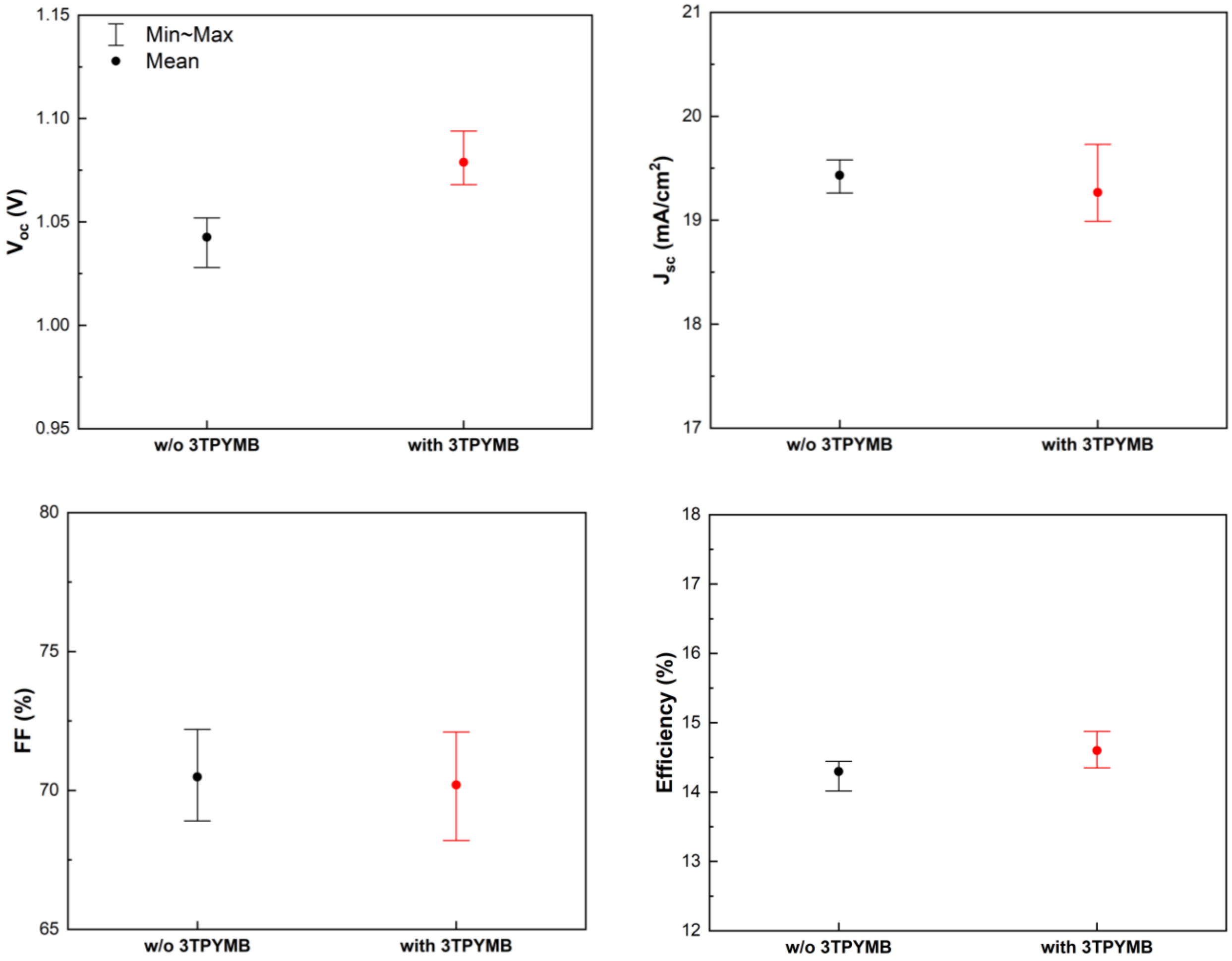

Figure S7. Comparison among PV parameters statistics of the flexible PSCs with and without 3TPYMB interfacial modification.

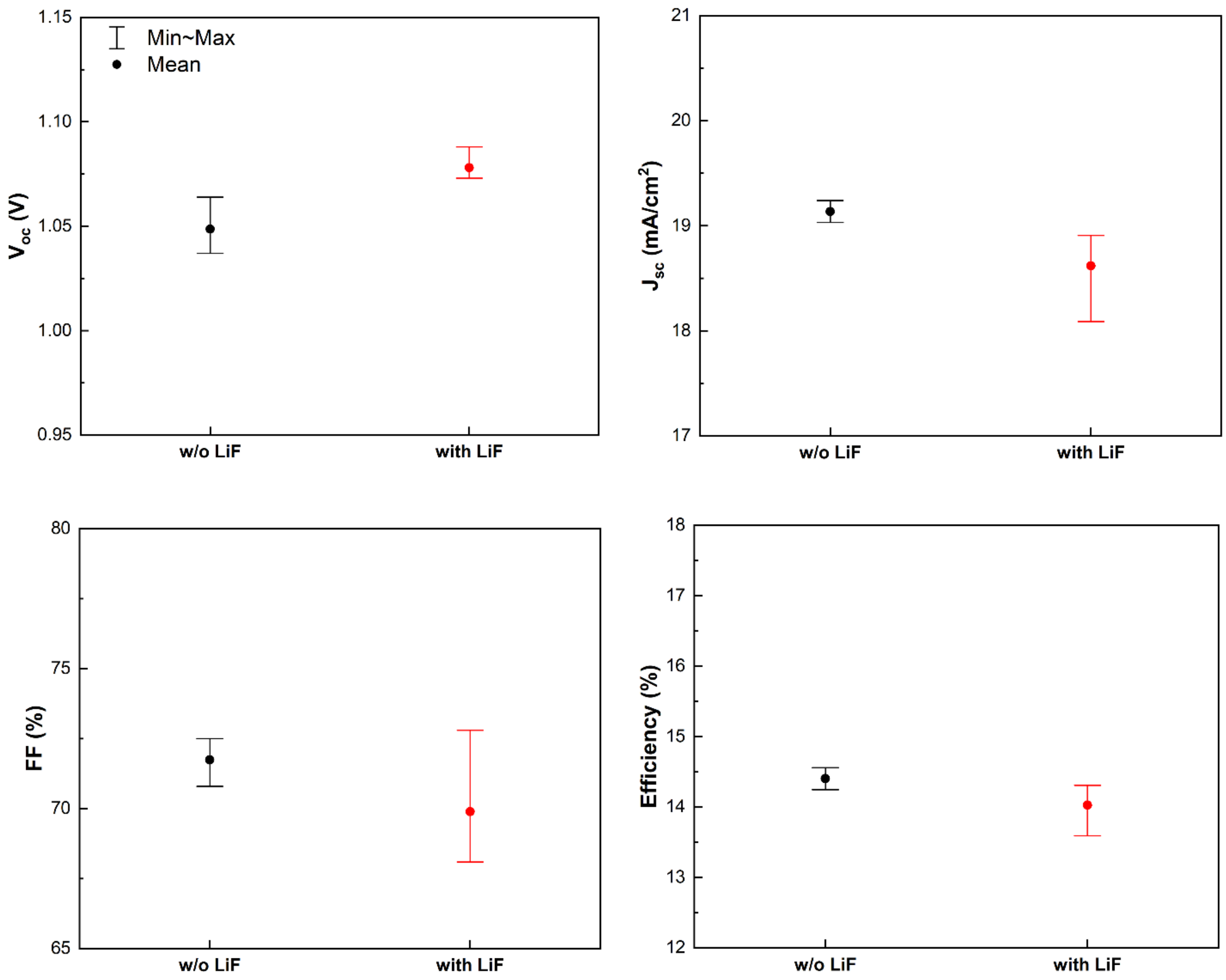

Figure S8. Comparison among PV parameters statistics of the flexible PSCs with and without LiF interlayer.

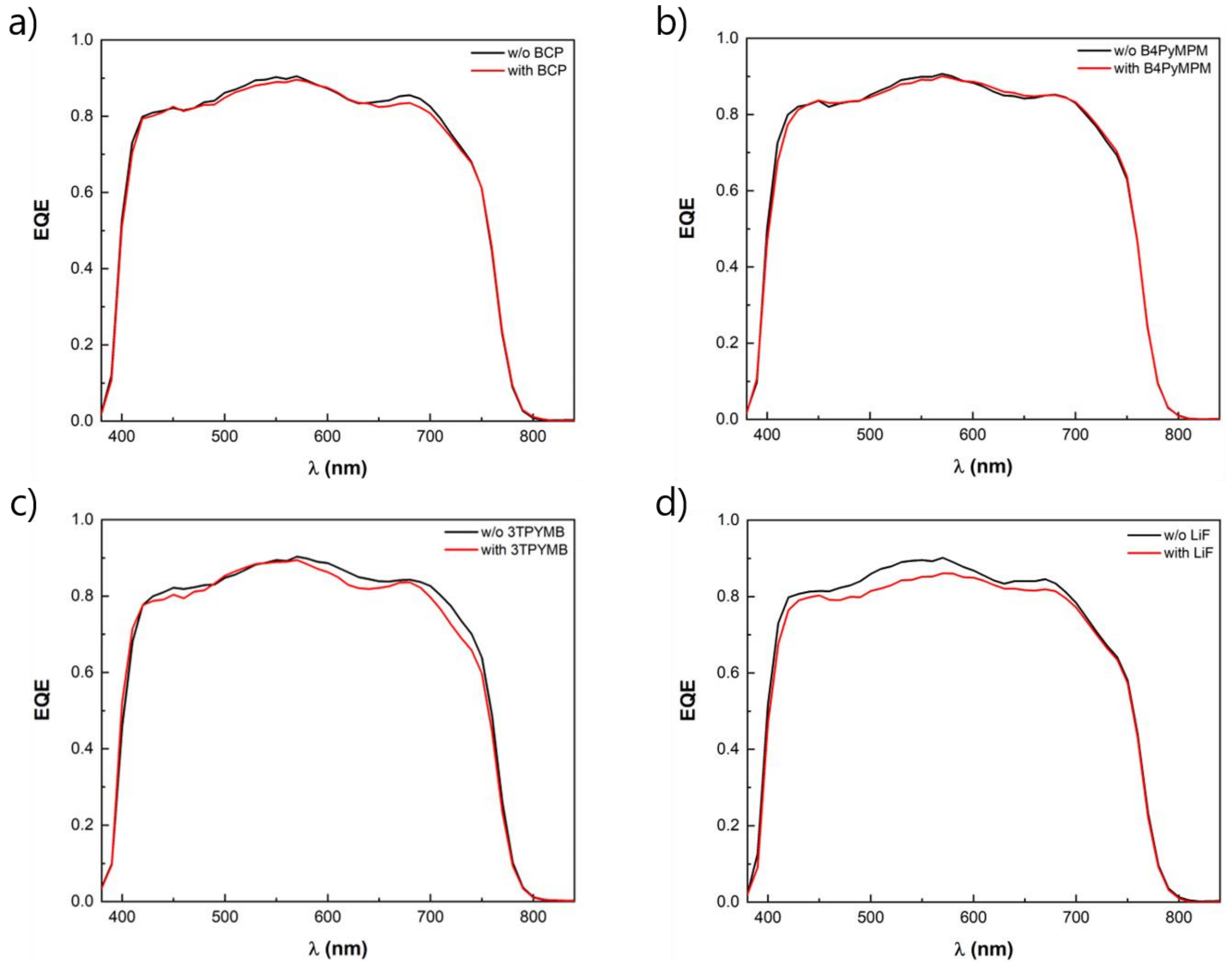

Figure S9. Comparison of EQE spectra for the different interfacial modifications investigated (devices shown in Figure 3). a) without BCP (extracted J_{sc}=19.5 mA/cm^2) and with BCP (J_{sc}=19.3 mA/cm^2), b) without B4PyMPM (J_{sc}=19.5 mA/cm^2) and with B4PyMPM (J_{sc}=19.5 mA/cm^2), c) without 3TPYMB (J_{sc}=19.5 mA/cm^2) and with 3TPYMB (J_{sc}=19.2 mA/cm^2), d) without LiF (J_{sc}=19.3 mA/cm^2) and with LiF (J_{sc}=18.7 mA/cm^2).

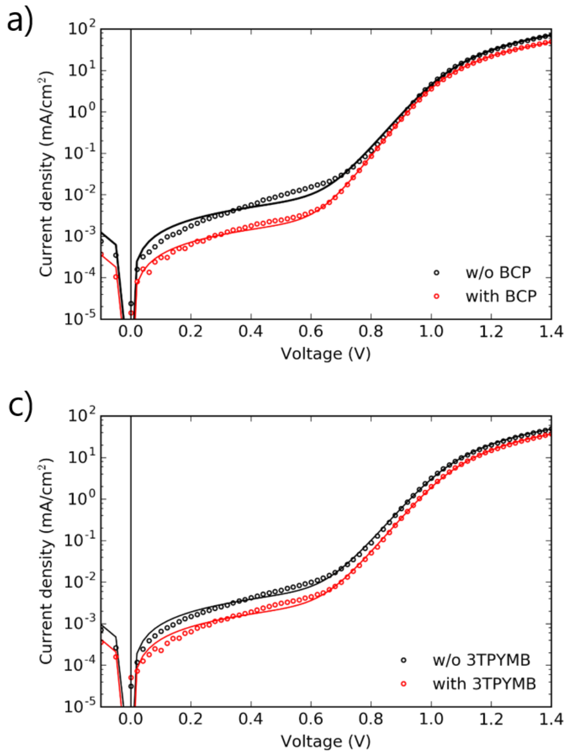

Figure S10. Comparison of semi-log plots of dark *J*-*V* curves of devices from Figure 3. a) with and without BCP, b) with and without B4PyMPM, c) with and without 3TPYMB, d) with and without LiF interfacial modifications.

Table S1. R_S values obtained from fitting dark *J-V* curves to a single diode with two resistors model.

	**w/o BCP**	**with BCP**	**w/o B4PyMPM**	**with B4PyMPM**	**w/o 3TPYMB**	**with 3TPYMB**	**w/o LiF**	**with LiF**
**R_s (Ωcm^2)**	3.8	6.2	3.3	6.8	5.5	7.0	5.6	7.6

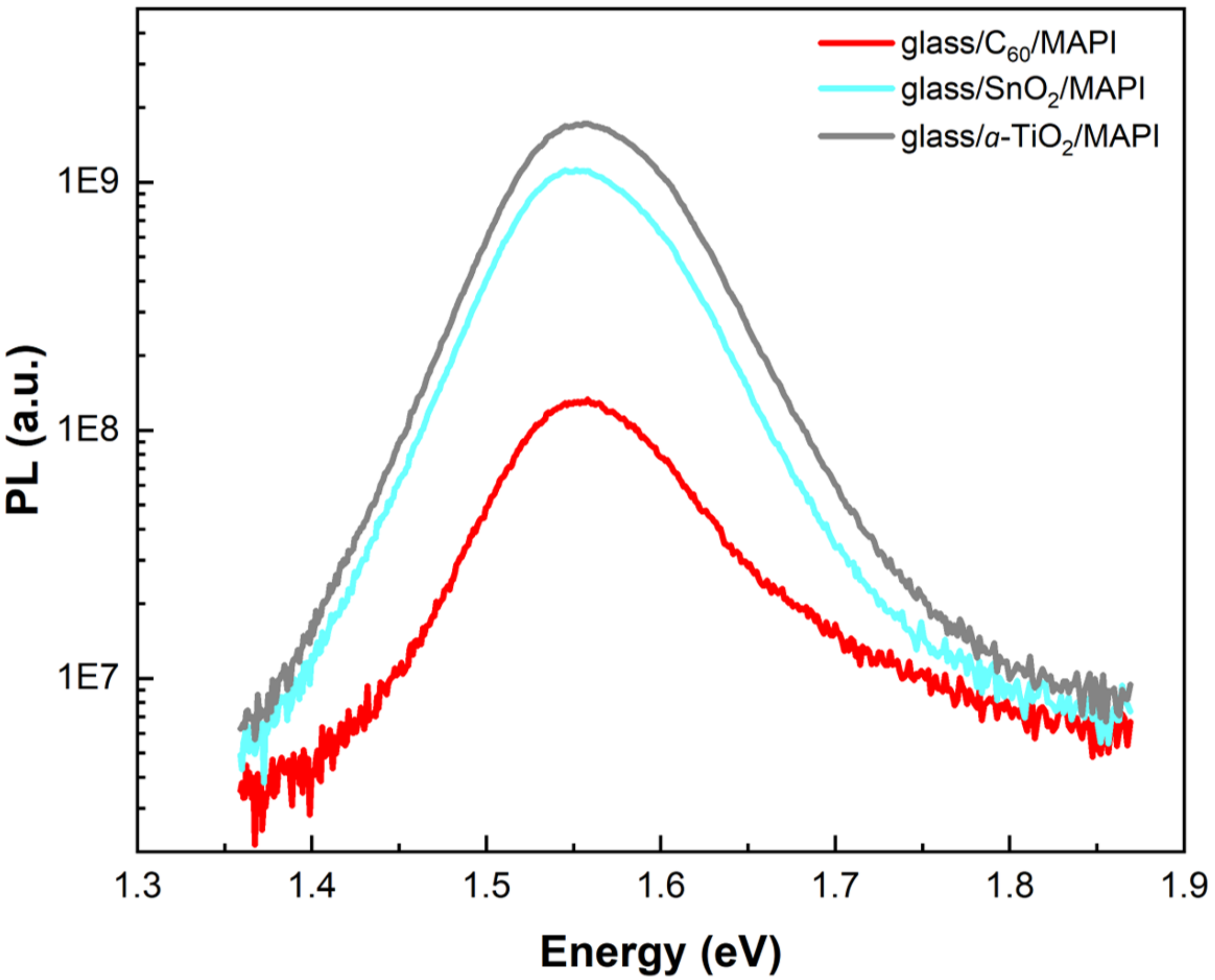

Figure S11. Comparison of PL spectra for different ETLs: glass/C_{60}/MAPI, glass/SnO_2/MAPI and glass/*a*-TiO_2/MAPI.

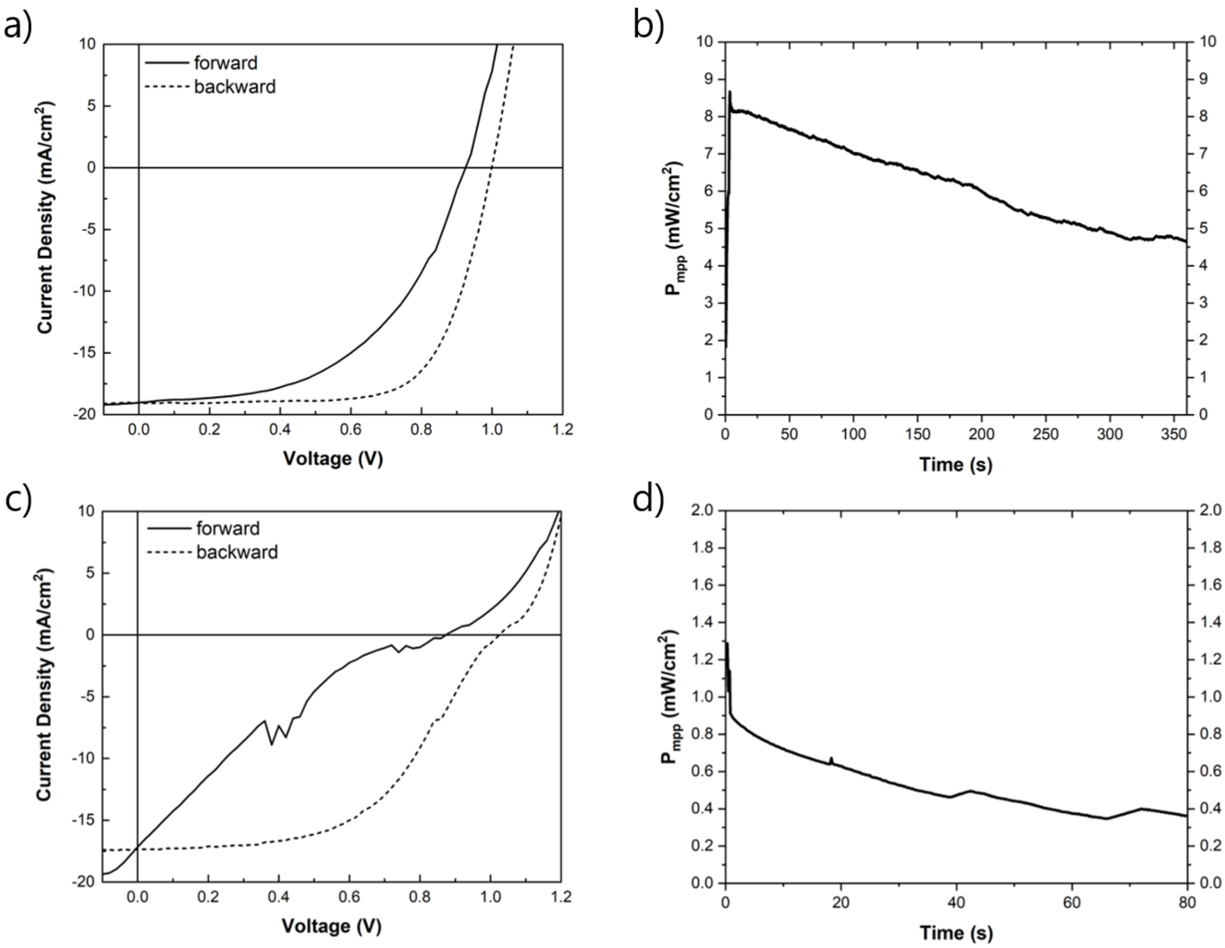

Figure S12. a) *J*-*V* characteristics for PSCs with spin coated SnO_2 as ETL (both forward and backward measurements), b) MPP tracking under 1 sun continuous illumination. c) *J*-*V* characteristics for PSCs with ALD deposited *a*-TiO_2 as ETL (both forward and backward measurements), d) MPP tracking under 1 sun continuous illumination.

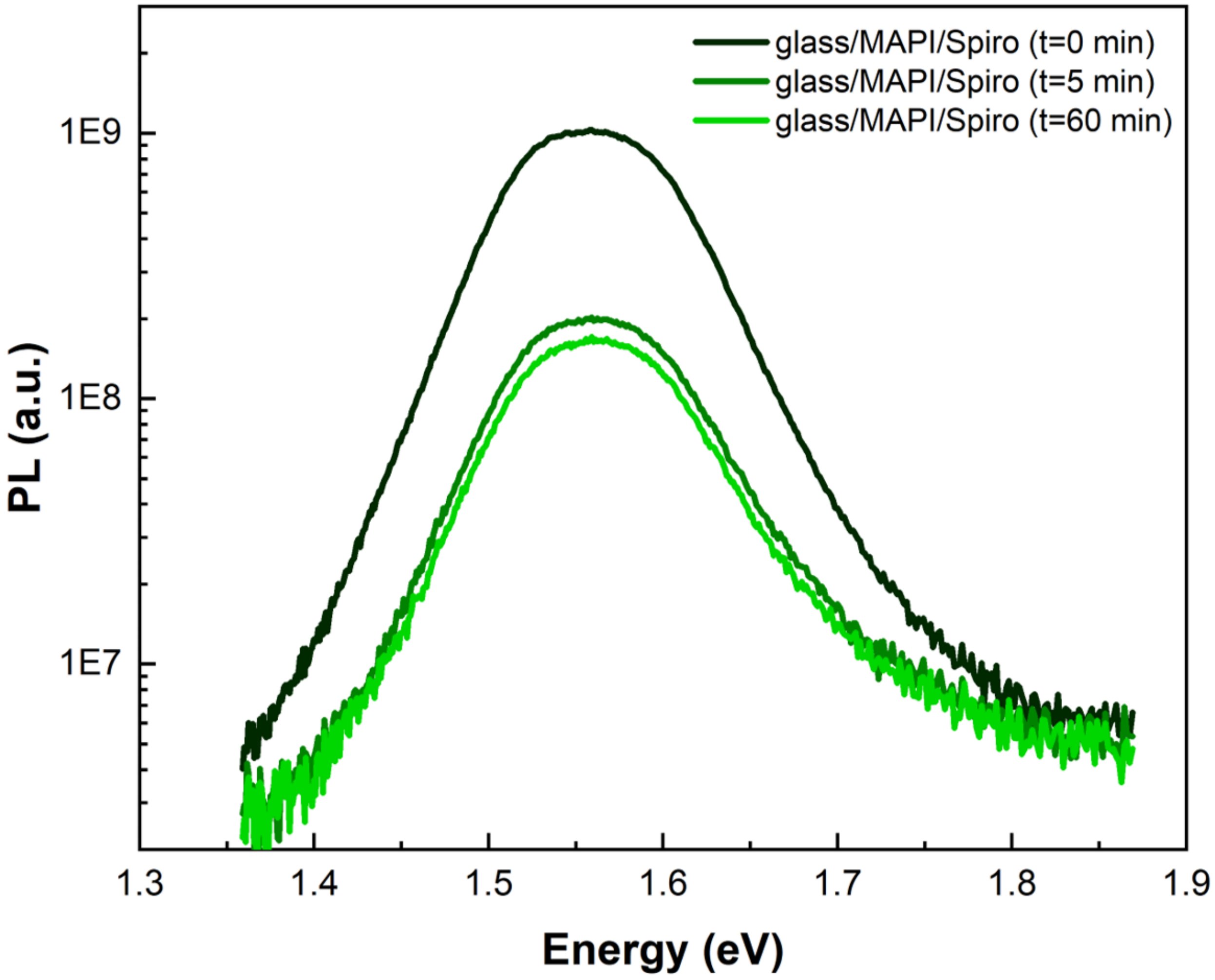

Figure S13. Comparison of PL spectra for glass/MAPI/Spiro at different exposure time to ambient atmosphere: t=0, 5 and 60 minutes.
